# LNP-mediated silencing of oncogenic lncRNA PVT1 enhances chemotherapeutic efficacy in osteosarcoma

**DOI:** 10.1016/j.bbrep.2026.102732

**Published:** 2026-08-19

**Authors:** Jiantong Wei, Zetao Qian, Qingqing Qin, Hao Chen, Wenqiang Liang, Ting Wang, Shengwan Wang, Wenji Wang, Yongping Wang, Yaohua Wang

**Affiliations:** a Department of Orthopedics, Zhangye People's Hospital Affiliated to Hexi University, Zhangye, Gansu, China; b Hexi University Institute Bone and Joint, Zhangye, Gansu, China; c Orthopedics Quality Control Center of Zhangye City, Zhangye, Gansu, China; d The First School of Clinical Medicine, Lanzhou University, Lanzhou, Gansu, China; e Gan Su ShengWan Biomedical Technology Co., Ltd. Zhangye, Gansu, China; f Department of Orthopedics, The First Hospital of Lanzhou University. Lanzhou, Gansu, China; g Department of Orthopedics, The Second Affiliated Hospital of Hainan Medical University, Haikou, Hainan, 570311, China

**Keywords:** Osteosarcoma, lncRNA PVT1, Lipid nanoparticles, RNA interference, Combination therapy

## Abstract

Osteosarcoma is an aggressive bone malignancy characterized by early metastasis, chemoresistance, and poor prognosis, particularly in recurrent or metastatic cases. The long non-coding RNA PVT1 has been implicated as an oncogene in various cancers, but its therapeutic potential in osteosarcoma remains underexplored. This study investigates the feasibility of targeting PVT1 using lipid nanoparticle-encapsulated siRNA (LNP-siPVT1) as a standalone or combination therapy for osteosarcoma. PVT1 expression was found to be significantly upregulated in osteosarcoma cell lines (U2OS, Saos-2, MG-63), patient tissues, and public datasets (GSE126209 and GSE309091). Tumor-targeted LNPs encapsulating siPVT1 were synthesized via microfluidic mixing and exhibited a mean diameter of ∼100 nm, favorable encapsulation efficiency, and accelerated siRNA release under acidic pH (5.5). In vitro, LNP-siPVT1 effectively suppressed PVT1 expression, reduced cell viability (IC_50_ = 29.30 nM), and induced apoptosis in MG-63 cells; moreover, combining LNP-siPVT1 with doxorubicin (DOX) or sorafenib produced synergistic cytotoxic and pro-apoptotic effects. In a humanized immune system mouse model bearing MG-63 xenografts, LNP-siPVT1 plus DOX significantly inhibited tumor growth compared to either monotherapy, without causing overt toxicity or body weight loss, as confirmed by histology and serum biochemistry. Collectively, PVT1 serves as a prognostic biomarker and a promising therapeutic target in osteosarcoma, and LNP-mediated delivery of siPVT1, especially in combination with conventional chemotherapeutics such as DOX, represents an effective and safe strategy to enhance anti-osteosarcoma efficacy, supporting further clinical translation of LNP-siPVT1-based therapy.

## Highlights

• LncRNA PVT1 is upregulated in osteosarcoma and linked to poor patient prognosis.
• LNP-encapsulated siPVT1 silences PVT1, inhibits proliferation and induces apoptosis.
• LNP-siPVT1 plus doxorubicin synergistically inhibits tumor growth with good biosafety.

## 1 Introduction

Osteosarcoma, the most prevalent primary malignant bone tumor in adolescents and young adults, presents a formidable clinical challenge due to its aggressive biological hallmarks, including rapid progression, early pulmonary metastasis, and intrinsic resistance to conventional therapeutic regimens[1,2]. Although multimodal therapeutic strategies integrating neoadjuvant chemotherapy, surgical resection, and adjuvant therapy have substantially improved clinical outcomes for patients with localized osteosarcoma, the prognosis for those with metastatic or recurrent disease remains dismal[3,4], with 5-year survival rates persisting below 30%[5]. This therapeutic plateau highlights the urgent imperative to identify novel molecular targets and develop innovative therapeutic approaches to overcome chemoresistance and impede metastatic dissemination[4,5].

PVT1 overexpression drives tumorigenesis across multiple cancer types by augmenting cellular proliferation, suppressing apoptosis, and facilitating epithelial-mesenchymal transition[[6], [7], [8]]. In osteosarcoma, both lncRAN PVT1 and its circular isoform, circPVT1, are significantly upregulated[9]. Functionally, they act as competing endogenous RNAs (ceRNAs) by sponging multiple tumor-suppressive microRNAs, including miR-195, miR-484, miR-205-5p, and miR-423-5p, thereby derepressing downstream oncogenic targets such as Cyclin D1, Bcl-2, HK2, Wnt5a/Ror2, c-FLIP, and FOXC2[[9], [10], [11], [12], [13]]. Through these regulatory networks, lncRNA PVT1 promotes tumor cell proliferation, inhibit apoptosis, reprogram glycolytic metabolism, enhance invasion and migration, and induce epithelial-mesenchymal transition[9,[14], [15], [16]]. Clinically, elevated expression of PVT1 transcripts correlates with advanced clinical stage, distant metastasis, and poor prognosis, highlighting their potential as diagnostic biomarkers and therapeutic targets in osteosarcoma[12,16].

RNA interference (RNAi)-based therapeutics present promising avenues for precision cancer treatment[17]. While small interfering RNA (siRNA) technology enables sequence-specific gene silencing, its clinical translation is hindered by substantial obstacles, including rapid nuclease degradation, inefficient cellular internalization, and non-specific biodistribution[18,19]. Lipid nanoparticles (LNPs) is a delivery platforms for nucleic acid therapeutics[[20], [21], [22], [23]]. These biomimetic systems confer multiple advantages: (1) enhanced serum stability via phospholipid encapsulation[24], (2) improved tumor accumulation through the enhanced permeability and retention (EPR) effect[25], and (3) pH-responsive drug release within the lysosome[26].

In the present study, we investigated the therapeutic potential of PVT1 silencing in osteosarcoma. Notably, PVT1 is highly expressed in osteosarcoma and correlates with poor prognosis. In vitro, PVT1 silencing via LNP-encapsulated siRNA (LNP-siPVT1) alone or combined with doxorubicin (DOX) effectively suppresses proliferation and induces apoptosis. We further demonstrate that PVT1 silencing via LNP-siPVT1, combination with DOX, exerts potent antitumor effects in a humanized immune system tumor mouse model. Collectively, these findings establish PVT1 as a promising therapeutic target in osteosarcoma and highlight the potential of LNP-siPVT1 delivery as an effective strategy to improve treatment outcomes.

## 2 Methods

### 2.1 Study approval

Adult NOD mice were obtained from Shouzheng Pharma Biotechnology Co., Ltd. (Wuhan, China) and housed under standard laboratory conditions (22 ± 3 °C, 12-h light/dark cycle) with ad libitum access to standard rodent chow. All animal experiments conducted in this study were reviewed and approved by the Ethics Committee of Zhangye Peoples Hospital Affiliated to Hexi University with the approval number: HFYER-B-202625.

### 2.2 Cell culture

The cell lines hFOB, Saos-2, MG-63 and U2OS used in this study were purchased from Procell (China), and cultured in DMEM medium containing 10% fetal bovine serum (FBS), 1% penicillin-streptomycin. These cells were cultured in a constant temperature incubator at 5% CO_2_ and 37 °C.

### 2.3 Western blotting

Cells were lysed using RIPA lysis buffer, and the protein concentration of the supernatant was determined using a BCA assay. Following electrophoresis, proteins were transferred onto 0.45 μm PVDF membranes. The membranes were then blocked with 5% non-fat milk for 1 h, incubated with primary antibodies over night, washed three times with TBST (10 min each), incubated with HRP-conjugated secondary antibodies for 1 h, and washed again three times with TBST (10 min each). Protein signals were visualized using an ECL chemiluminescence system.

### 2.4 Quantitative polymerase chain reaction (qPCR)

Total cellular RNA was extracted using TRIzol reagent (G3013, Servicebio). The extracted RNA was reverse transcribed according to the manufacturer's instructions using the reverse transcription kit (K1609, Thermo Scientific), followed by qPCR using the ChamQ Universal SYBR qPCR Master Mix (Q711-02, Vazymes, Nanjing, China) with a two-step PCR protocol. Gene expression fold changes were calculated using the 2^−^ΔΔCt method. The primer sequences used were as follows:

PVT1-F: TGAGAACTGTCCTTACGTGACC, PVT1-R: AGAGCACCAAGACTGGCTCT; ACTB-F: ATGTGCAAGGCCGGTTTCGC, ACTB-R: TACGAGTCCTTCTGGCCCAT.

### 2.5 Cell viability assay

Cells were seeded into 96-well plates at a density of 5 × 10^3^ cells per well and cultured overnight to allow proper attachment. The following day, the cells were treated with the indicated concentrations of drugs or vehicle control for 48 h. After treatment, the culture medium was replaced with 100 μL of fresh DMEM containing 0.5 mg/mL MTT (M5655, Sigma-Aldrich), and the plates were incubated at 37 °C for 2 h in a humidified incubator. Subsequently, the MTT-containing medium was carefully aspirated, and the intracellular formazan crystals were dissolved in 100 μL of dimethyl sulfoxide (DMSO) per well. The optical density (OD) was then recorded at 570 nm using a BioTek Synergy H1 microplate reader, with a reference wavelength of 630 nm for background correction.

### 2.6 Apoptosis assay

After treatment, both adherent and floating cells were harvested, washed twice with cold PBS, and resuspended in 1× binding buffer. Apoptosis was detected using the Annexin V-FITC/7-AAD apoptosis detection kit (E-CK-A212, Elabscience) according to the manufacturer's instructions. Stained cells were immediately analyzed on a CytoFLEX flow cytometer (Beckman Coulter). The total apoptotic rate was calculated as the sum of early and late fractions. Data acquisition and analysis were performed using FlowJo software (v10, FlowJo LLC). All experiments were carried out in triplicate.

### 2.7 LNP-siRNA assembly

Lipid nanoparticles (LNPs) were prepared using a microfluidic mixing method. The lipid mixture comprised DLin-MC3-DMA, porphyrin-lipid, cholesterol, and DMG-PEG2000 at a molar ratio of 50:10:38.5:1.5, dissolved in anhydrous ethanol. siRNA targeting PVT1 (siPVT1, sequence: 5′-CCCAACAGGAGGACAGC-3′) was dissolved in 25 mM sodium acetate buffer (pH 4.0). The aqueous and ethanolic phases were combined at a flow rate ratio of 3:1 (aqueous:ethanol) using herringbone microfluidic mixing chips (Microfluidic ChipShop, Jena, Germany), with a total flow rate of 10 mL min^−1^. The resulting LNP dispersion was dialyzed against phosphate-buffered saline (PBS, pH 7.4) overnight at 4 °C using a Slide-A-Lyzer dialysis cassette (Thermo Fisher Scientific, Waltham, MA, USA) to remove residual ethanol and exchange the buffer. The LNPs were then concentrated using Amicon® Ultra centrifugal filters (MWCO 100 kDa, Merck Millipore, Billerica, MA, USA) and sterilized by passage through a 0.22 μm polyethersulfone membrane filter (Millipore, Burlington, MA, USA). All prepared LNPs were stored at 4 °C and used within 48 h.

### 2.8 LNP particle size and zeta potential detection

LNP-siPVT1 samples were diluted in deionized water to a final concentration of 0.1% (w/w) and sonicated in a water bath sonicator (Fisher Scientific, Pittsburgh, PA, USA) for 3 min to obtain a homogeneous dispersion. The average hydrodynamic diameter, polydispersity index (PDI), and zeta potential were measured using a Zetasizer Nano-ZS90 instrument (Malvern Instruments Ltd., Malvern, UK) at 25 °C. Prior to each measurement, samples were equilibrated for 2 min.

### 2.9 In vitro cumulative release of LNP-siPVT1

The in vitro release profile of LNP-siPVT1 was evaluated using a dialysis method. Dialysis bags with a molecular weight cutoff of 100 kDa (Spectra/Por®, Spectrum Laboratories, Rancho Dominguez, CA, USA) were pretreated by soaking in deionized water for 30 min prior to use. Briefly, 1 mL of LNP-siPVT1 dispersion was placed into the pretreated dialysis bag, which was then immersed in 50 mL of release medium (deionized water or phosphate-buffered saline, PBS) at pH 5.5, 6.5, or 7.4. All samples were incubated in an orbital shaker at 100 rpm and 37 °C. At designated time intervals (0, 1, 2, 4, 6, 8, 12, 24, and 48 h), 1 mL of the external medium was withdrawn and replaced with an equal volume of fresh pre-warmed medium to maintain sink conditions. The concentration of siPVT1 in the withdrawn samples was determined using a NanoDrop 2000 spectrophotometer (Thermo Fisher Scientific, Waltham, MA, USA) by measuring the absorbance at 260 nm. The cumulative release percentage was calculated relative to the total amount of encapsulated siRNA, which was determined by lysing an equal volume of LNP-siPVT1 with 1% Triton X-100.

### 2.10 Reconstitution of human immune system and subcutaneous tumor xenograft model

NOD mice were used for the reconstitution of human immune system. Human peripheral blood mononuclear cells (PBMCs) were purchased from Milesbio (Shanghai, China). A total of 1 × 10^7^ PBMCs suspended in 200 μL of sterile PBS were injected into each mouse via the lateral tail vein. At 4 weeks post-transplantation, peripheral blood samples were collected from the retro-orbital sinus, and the proportion of human CD45^+^ cells among total leukocytes was determined by flow cytometry using PE-conjugated anti-human CD45 antibody (12-0459-42, eBioscience). Mice with >25% human CD45^+^ cells were considered successfully reconstituted and used for subsequent experiments.

For subcutaneous tumor xenograft studies, MG63 human osteosarcoma cells in the logarithmic growth phase were harvested, washed twice with PBS, and resuspended in serum-free DMEM. A total of 1 × 10^7^ cells in 100 μL were subcutaneously injected into the right flank of the reconstituted NOD mice. Five days after tumor inoculation, mice were randomly allocated into five groups (n = 5 per group) and treated as follows: (1) siGFP control (20 μg per mouse), (2) siPVT1 (20 μg per mouse), (3) LNP-siPVT1 (20 μg siRNA equivalent per mouse), (4) doxorubicin (DOX, 5 mg kg^−1^, S1208, Selleck), and (5) LNP-siPVT1 + DOX (same doses and routes as above). The LNP formulations and siRNAs were administered via the tail vein twice per week, whereas DOX was given via intraperitoneal injection once per week. Tumor dimensions were measured with a digital caliper twice weekly, and tumor volume was calculated using the formula: V = (L × W^2^)/2, where L is the longest diameter and W is the perpendicular width. After 4 weeks of treatment, all mice were euthanized and sacrificed, and the subcutaneous tumors were excised, weighed, and photographed. Major organs (heart, liver, spleen, lung and kidney) were also collected, fixed in 4% paraformaldehyde (PFA), and processed for further histopathological analysis.

### 2.11 H&E staining

Heart, liver, spleen, lung, and kidney tissues were fixed overnight at 4 °C in 4% PFA. Following fixation, tissues were sequentially dehydrated and embedded. The paraffin-embedded tissues were sectioned into 5 μm-thick slices. Sections were mounted on glass slides, deparaffinized and rehydrated by distilled water. For histological examination, sections were stained with hematoxylin (G1004, Servicebio) for 3 min at room temperature. Subsequently, sections were counterstained with eosin (G1001, Servicebio) for 10 s. Stained sections were scanned using a digital slide scanner (LG-S80, Servicebio).

### 2.12 Serum biochemical detection

Whole blood samples were centrifuged at 2000 g for 10 min at 4 °C, and the supernatant serum was collected. Subsequently, serum levels of AST (C010-2-1, njjcbio), ALT (C009-2-1, Njjcbio), blood urea nitrogen (BUN, C013-2-1, Njjcbio), and creatinine (Cr, C011-2-1, Njjcbio) were measured in accordance with the manufacturers protocols provided by Nanjing Jiancheng Bioengineering Institute.

### 2.13 Statistical analysis

The statistical analyses were conducted using Graphpad Prism 10.1 software. The significance level among two groups was calculated using two-tailed unpaired *t*-test. Statistical significance of more than two groups was assessed by the analysis of variance (ANOVA), followed by a Tukey post hoc test. *p*-value < 0.05 was considered to show a significant difference. All experiments were repeated at least three times to ensure reproducibility, and representative results are shown.

## 3 Results

### 3.1 Aberrant upregulation of PVT1 in osteosarcoma is associated with poor prognosis

**Fig. 1:**
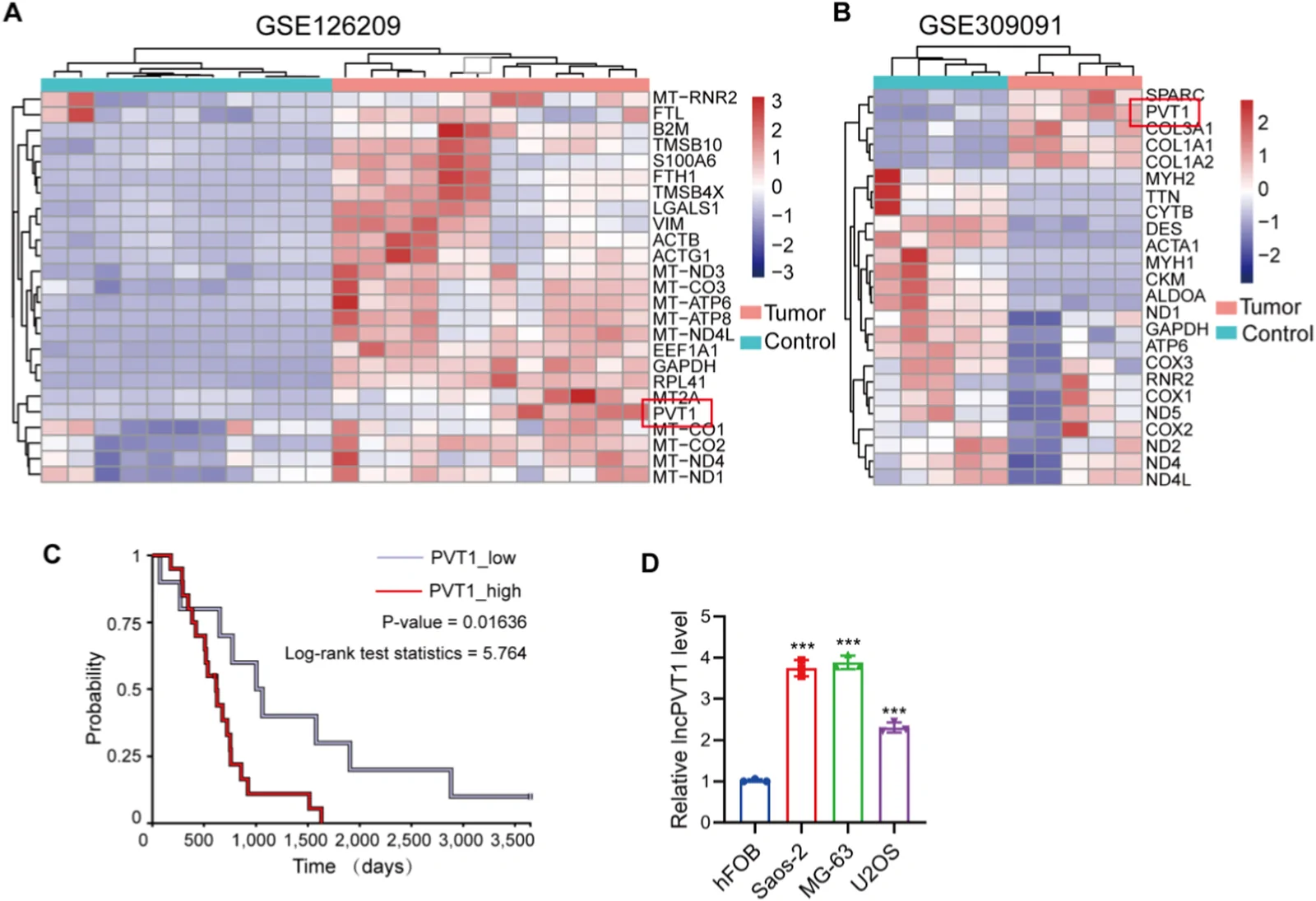
PVT1 is upregulated in osteosarcoma and associated with poor prognosis. (A-B) The heatmap exhibited the significantly differentially expressed genes between normal and tumor from GSE126209 (A) and GSE309091 (B)·(C) Kaplan-Meier overall survival curve of PVT1 expression in osteosarcoma patients was analyzed by the UCSC Xena web platform. The red curve represented patients with high PVT1 expression, while the blue curve indicated patients with low PVT1 expression. (D) qPCR analysis of PVT1 expression in hFOB, MG-63, U2OS and Saos-2 cell lines (n = 3). Data are expressed as mean ± SD, statistical significance was assessed by two-tailed Students t-test. *p < 0.05, ***p < 0.001.

Analysis of RNA sequencing data from the GEO database (GSE126209 and GSE309091) revealed a marked upregulation of PVT1 in osteosarcoma tumor compared to adjacent tissues (Fig. 1A-B). Kaplan-Meier survival analysis further demonstrated that elevated PVT1 expression was significantly associated with poorer overall survival in osteosarcoma patients (Fig. 1C). These bioinformatic findings were experimentally corroborated by quantitative real-time PCR (qPCR) analysis, which showed consistently higher PVT1 expression in three human osteosarcoma cell lines (Saos-2, MG-63, and U2OS) relative to the murine pre-osteoblastic cell line hFOB (Fig. 1D). Collectively, these results establish that PVT1 is aberrantly overexpressed in osteosarcoma and suggest its potential utility as both a prognostic biomarker and a therapeutic target.

### 3.2 Synthesis and characterization of LNP-siPVT1

**Fig. 2:**
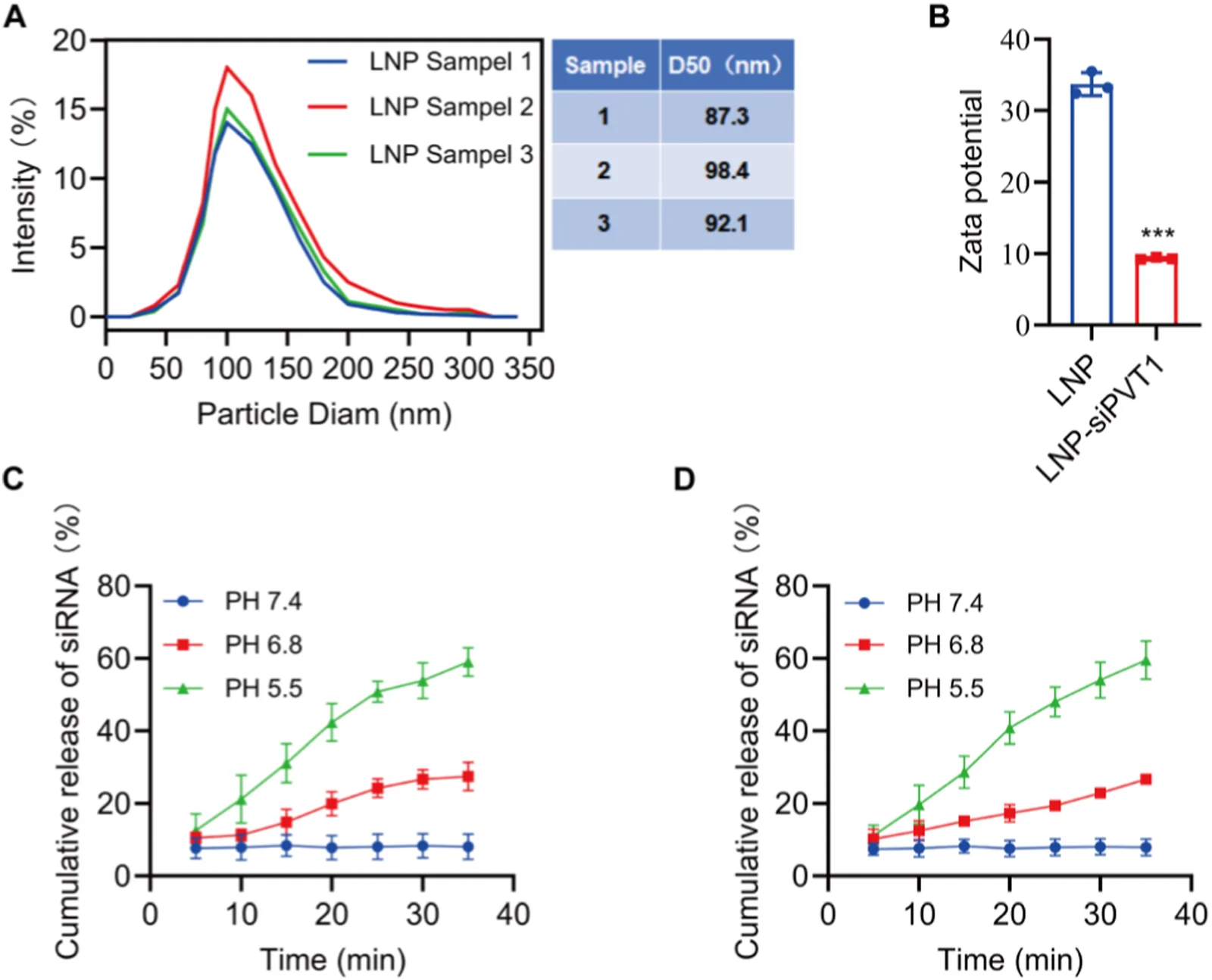
Characterization and pH-responsive siRNA release profiles of LNP formulations.(A) Particle size distribution profiles of the LNP formulations (LNP Sample 1, LNP Sample 2, LNP Sample 3). The D50 values (median particle size) are indicated in the table below. (B) The Zeta potential of LNP and LNP-siPVT1 were calculated by Zetasizer Nano-ZS90. (C-D) The cumulative release curves of LNP in PBS (A) and FBS (B) over time under different pH condition. Data are expressed as mean ± SD (n = 3), statistical significance was assessed by two-tailed Students t-test. ***p < 0.001.

We next investigated whether targeted silencing of PVT1 using LNP-encapsulated siRNA could exert antitumor effects in osteosarcoma. LNP is an advanced drug delivery system used for transporting nucleic acids[27]. To assess the therapeutic potential of PVT1 silencing in osteosarcoma, we fabricated LNP-siPVT1 using a microfluidic mixing approach and characterized its physicochemical properties, including particle size, zeta potential, and siRNA release profile. Dynamic Light Scattering (DLS) revealed that the LNP-siPVT1 formulation maintained a uniform and stable hydrodynamic diameter centered at about 100 nm (D50 range: 87.3-98.4 nm), a dimension well-suited for passive tumor accumulation via the enhanced permeability and retention (EPR) effect (Fig. 2A). A significant decrease in zeta potential was observed for LNP-siPVT1 compared to unloaded LNPs, providing evidence for efficient siRNA loading (Fig. 2B). Solid tumor tissues establish an acidic extracellular microenvironment (pH 6.5-7.0), which is substantially reduced relative to the physiological pH of normal tissues (pH 7.4) yet considerably elevated compared to the intralysosomal pH (approximately 4.5-5.5)[28,29]. To assess siRNA release from LNP-siPVT1 under physiologically relevant pH conditions, we conducted in vitro release studies and monitored the cumulative release of siPVT1 over time. The resulting release profiles revealed a marked pH-dependent release pattern, with significantly enhanced siRNA liberation observed as the environmental pH decreased from 7.4 to 5.5 in both PBS and FBS media (Fig. 2C-D). In conclusion, LNP-siPVT1 was successfully formulated and exhibits desirable physicochemical characteristics alongside robust pH-responsive release kinetics, underscoring its promise as an effective nucleic acid delivery platform for osteosarcoma treatment.

### 3.3 LNP-siPVT1 suppresses osteosarcoma cell progression and enhances chemosensitivity in vitro

**Fig. 3:**
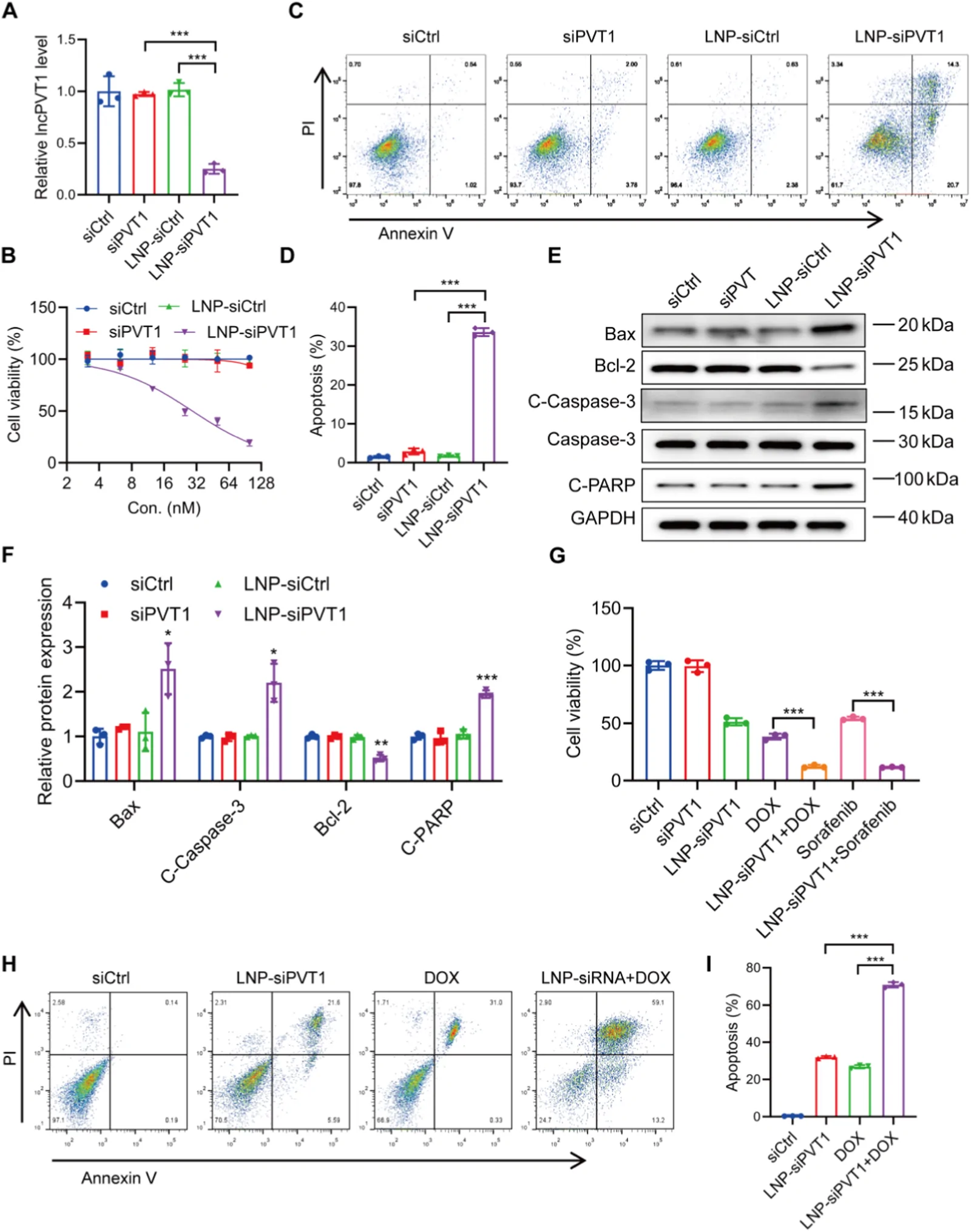
In vitro anticancer effects of LNP-siPVT1 in MG-63 osteosarcoma cells. (A) Relative expression of PVT1 in MG-63 cells treated with siCtrl, free siPVT1, LNP-siCtrl, or LNP-siPVT1 for 48 h, measured by qPCR. (B) Cell viability curves of MG-63 cells after treatment with the indicated formulations for 48 h, determined by MTT assay. (C-D) Representative flow cytometry plots (C) and quantitative analysis (D) of Annexin V/7-AAD staining showing apoptosis induction in MG-63 cells treated with siCtrl, siPVT1, LNP-siCtrl, or LNP-siPVT1 for 48 h. (E-F) MG-63 cells were treated with siCtrl, siPVT1, LNP-siCtrl, or LNP-siPVT1 for 48 h. Western blot analysis the protein expression (E) and quantification (F). (G) Cell viability of MG-63 cells treated with LNP-siPVT1, doxorubicin (DOX), sorafenib, or their combinations for 48 h. (H-I) Representative flow cytometry plots (H) and quantitative analysis (I) of Annexin V/7-AAD staining showing apoptosis induction in MG-63 cells treated with the combination of LNP-siPVT1 and DOX compared to monotherapies. All data are expressed as mean ± standard deviation (SD, n = 3). Statistical significance was analyzed by one-way ANOVA; ***p < 0.001.

We next investigated its biological effects on osteosarcoma cells in vitro. We treated MG-63 tumor cells with siGFP (siCtrl), LNP, free siPVT1 or LNP-siPVT1. qPCR analysis revealed that LNP-siPVT1 significantly downregulated PVT1 expression compared with free siPVT1 or LNP alone (Fig. 3A). MTT assay showed LNP-siPVT1 exhibited potent cytotoxicity against MG-63 cells, with an IC_50_ of 29.30 nM (Fig. 3B). Moreover, LNP-siPVT1 markedly induced apoptosis in MG-63 cells (Fig. 3C-D). To further elucidate the mechanisms underlying LNP-siPVT1 induced apoptosis, we performed Western blot analysis to examine the expression of key apoptotic regulators. Treatment with LNP-siPVT1 significantly upregulated the expression of the pro-apoptotic protein Bax, while concomitantly downregulating the anti-apoptotic protein BCL2 compared with the siCtrl, LNP, and free siPVT1 groups (Fig. 3E-F). Collectively, these data indicate the anticancer potential of LNP-siPVT1. Moreover, we evaluated the therapeutic efficacy of combining LNP-siPVT1 with the chemotherapeutic agents sorafenib or doxorubicin (DOX). Cell viability assays demonstrated that combination therapies of LNP-siPVT1 with sorafenib or DOX exhibited superior anticancer efficacy compared to monotherapies (Fig. 3G). Furthermore, flow cytometry analysis of apoptosis indicated that combining LNP-siPVT1 with DOX significantly enhanced apoptosis in MG-63 cells compared to monotherapies (Fig. 3H-I). Taken together, LNP-siPVT1 exhibits potent anticancer activity and demonstrates synergistic effects with multiple chemotherapeutic agents.

### 3.4 LNP-siPVT1 combined with DOX suppresses osteosarcoma growth in a humanized immune system mouse model

**Fig. 4:**
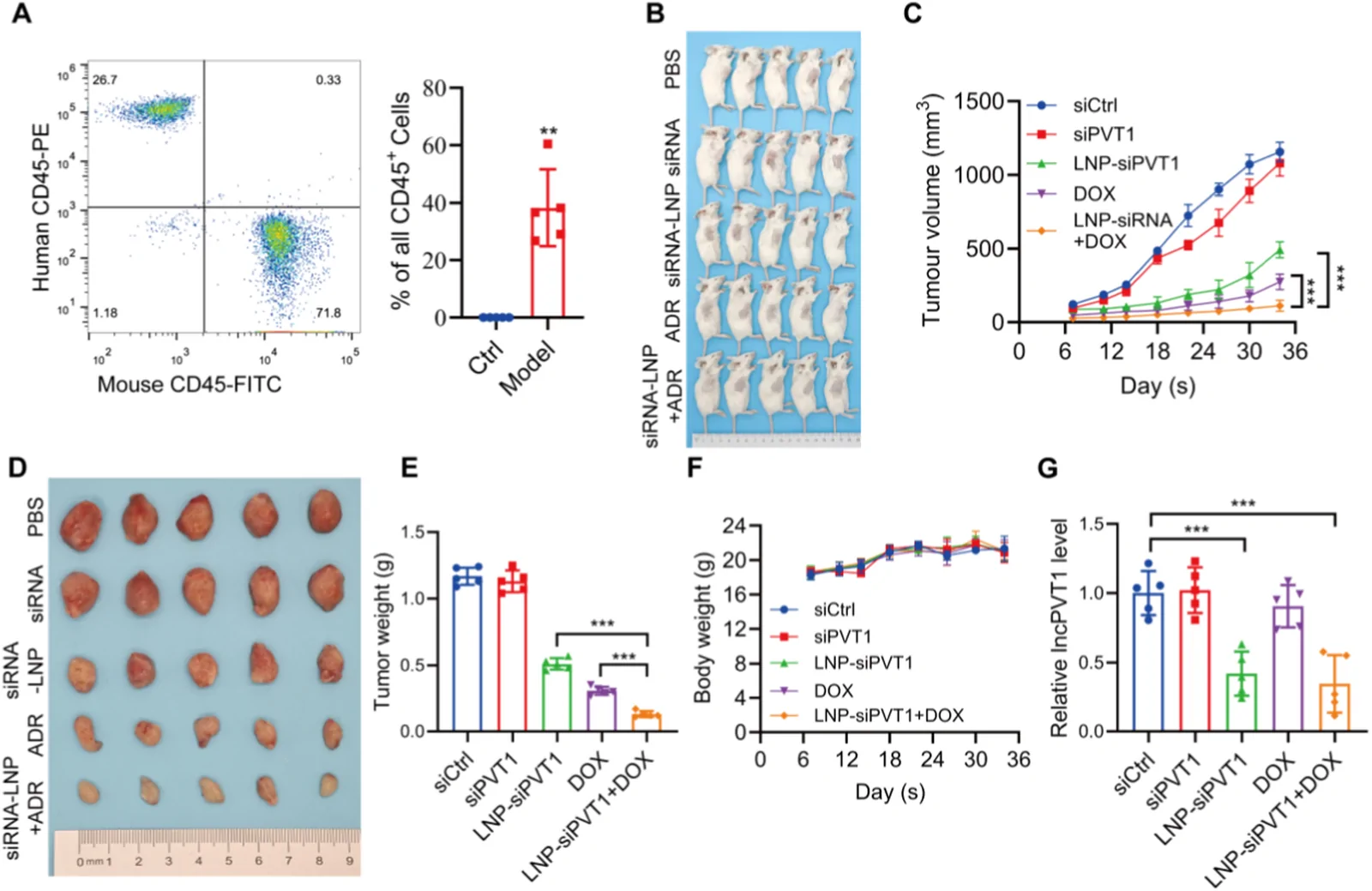
LNP-siPVT1 synergizes with doxorubicin to inhibit osteosarcoma growth in a humanized immune mouse model. (A) Flow cytometry analysis of human CD45^+^ cell reconstitution in the peripheral blood of NOD mice after human PBMC transplantation. Mice with >25% human CD45^+^ cells were selected for subsequent experiments. (B) Representative photographs of humanized mice bearing MG-63 xenograft tumors at the endpoint of treatment. (C) Tumor volume growth curves of mice treated with siCtrl, siPVT1, LNP-siPVT1, DOX, or LNP-siPVT1+DOX (n = 5 per group). (D) Representative images of tumors from each treatment group at the endpoint. (E) Tumor weights from each treatment group at the endpoint. (F) Body weight changes of mice during treatment. (G) qPCR analysis of PVT1 expression in tumor tissues from each treatment group at the endpoint. All data are expressed as mean ± standard deviation (SD). Statistical significance was analyzed by one-way ANOVA; **p < 0.01, ***p < 0.001.

To further elucidate the anti-tumor effect of LNP-siPVT1 in vivo, we reconstructed the human immune system in NOD mice through intravenous injection of human PBMC. Four weeks after PBMC transplantation, flow cytometry analysis of human CD45^+^ cell proportion in peripheral blood was performed, and mice with a proportion exceeding 25% were selected (Fig. 4A). Then we subcutaneously injected MG63 cells into these mice. After 5 days, the mice were randomly divided into five groups and treated with siCtrl, siPVT1, LNP-siPVT1, DOX or the combination of LNP-siPVT1 and DOX (LNP-siPVT1+DOX). Tumor volume and endpoint weight analyses demonstrated that LNP-siPVT1 combined with DOX exerted the most potent antitumor effect (Fig. 4B–E). All treatment regimens were well tolerated, with no significant weight body loss (Fig. 4F). qPCR analysis further verified that LNP-siPVT1 effectively downregulated PVT1 expression in tumor tissues (Fig. 4G). In summary, LNP-siPVT1 effectively silenced PVT1 expression in tumor tissues and, when combined with DOX, exerted the potent antitumor efficacy, and represented a promising therapeutic strategy for osteosarcoma in a humanized immune environment.

**Fig. 5:**
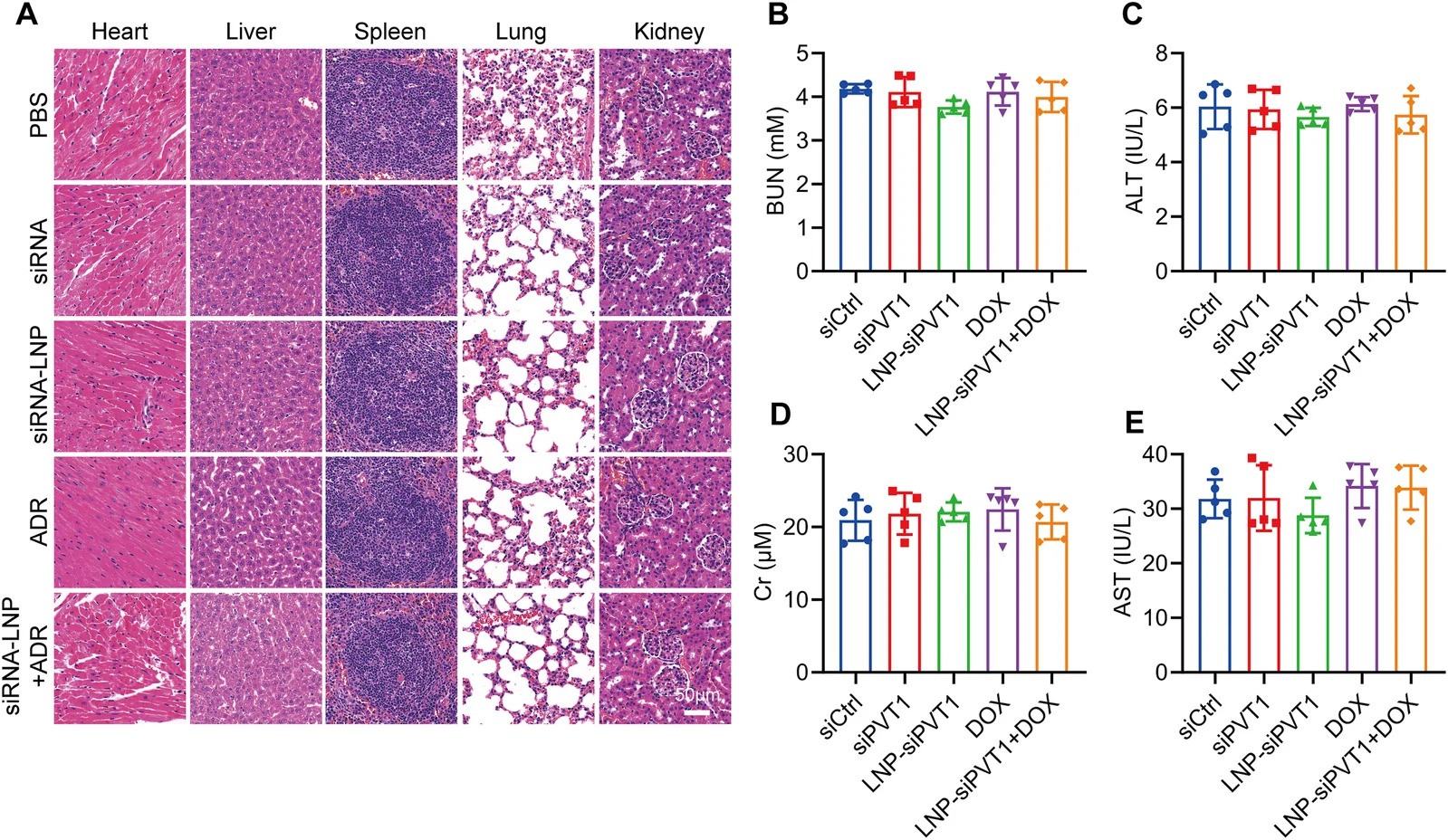
Biosafety evaluation of LNP-siPVT1 combined with DOX in vivo. (A) H&E staining of the heart, liver, spleen, lung and kidney from mice treated with siCtrl, siRNA, LNP-siPVT1, DOX and LNP-siPVT1+DOX. (B-E) The concentration of BUN (B), ALT (C), Cr (D) and AST (E) in the serum were detected from siCtrl, siRNA, LNP-siPVT1, DOX and LNP-siPVT1+DOX treated NOD mice.

To determine the safety profiles of the therapeutic regimens, organ toxicity was evaluated. H&E staining demonstrated preserved tissue architecture without pathological alterations in the liver, spleen, lung, and kidney upon LNP-siPVT1 or DOX treatment (Fig. 5A). Serum biochemical analysis of AST, ALT, blood urea nitrogen (BUN) and creatinine (Cr) further confirmed there was no damage in liver and kidney upon treatments, indicating that the treatments had no significant toxicity (Fig. 5B).

## 4 Discussions

In the present study, we have made several key discoveries: (1) the oncogenic lncRNA PVT1 is aberrantly overexpressed in osteosarcoma cell lines, patient tissues, and public datasets, and its elevated expression correlates with poor prognosis; (2) LNP-encapsulated siPVT1, fabricated via microfluidic mixing, exhibits favorable physicochemical characteristics-mean diameter ∼100 nm, high encapsulation efficiency, and pH-responsive release kinetics-that enable efficient PVT1 silencing in vitro and in vivo; (3) LNP-siPVT1 significantly suppresses cell viability (IC_50_ = 29.30 nM) and induces apoptosis in MG-63 cells, and synergistically enhances the cytotoxic effects of doxorubicin and sorafenib; (4) in a humanized immune system mouse model bearing MG-63 xenografts, combination treatment with LNP-siPVT1 and doxorubicin achieves superior inhibition of tumor growth without notable systemic toxicity or organ impairment. Collectively, these observations establish PVT1 as a viable therapeutic target in osteosarcoma and demonstrate that LNP-mediated delivery of siPVT1, particularly in combination with conventional chemotherapy, represents a safe and effective strategy to overcome chemoresistance and improve treatment outcomes.

Osteosarcoma remains a devastating malignancy, particularly for patients with metastatic or recurrent disease, for whom 5-year survival rates have stagnated below 30% despite decades of clinical trials[4,5]. The therapeutic plateau achieved with conventional chemotherapy underscores the urgent need for novel molecularly targeted strategies. PVT1 has emerged as a critical oncogenic driver across multiple cancer types, functioning as a competing endogenous RNA (ceRNA) that sponges tumor-suppressive microRNAs and derepresses downstream oncogenic targets[[6], [7], [8]]. In osteosarcoma, the dual roles of linear PVT1 and its circular isoform circPVT1 highlight their function in sponging miR-195, miR-484, miR-205-5p, and miR-423-5p, thereby upregulating oncogenic effectors such as Cyclin D1, Bcl-2, HK2, Wnt5a/Ror2, and FOXC2[9]. Our findings corroborate these mechanistic insights by demonstrating that PVT1 is markedly overexpressed in osteosarcoma cell lines and patient tissues, and that its knockdown significantly downregulates Bcl-2 expression, thereby promoting apoptosis.

While previous studies have predominantly focused on the molecular circuitry of PVT1-mediated oncogenesis, our work advances the field by translating these mechanistic observations into a therapeutically actionable strategy. Specifically, we have successfully encapsulated siPVT1 into a clinically relevant LNP formulation that addresses the critical delivery barriers-rapid nuclease degradation, inefficient cellular internalization, and non-specific biodistribution-that have historically hindered RNAi-based therapies[18,19]. In this regard, our study parallels observations in breast cancer studies, which have also proposed PVT1 as a therapeutic target and emphasized the need for efficient delivery systems[30]. The ∼100 nm particle size further facilitates passive tumor accumulation via the enhanced permeability and retention (EPR) effect, a hallmark of solid tumor vasculature[25]. The pH-responsive release profile of LNP-siPVT1 showing accelerated siRNA liberation under acidic conditions (pH 5.5) is biologically advantageous, as the acidic tumor microenvironment (pH 6.5-7.0) and the endolysosomal compartment (pH 4.5-5.5) can trigger localized siRNA release, enhancing target gene silencing specifically within tumor cells while minimizing off-target effects[28,29]. Therefore, our work not only validates PVT1 as a therapeutic target but also provides a potential delivery platform.

Chemoresistance, particularly to doxorubicin, remains a major obstacle in the clinical management of osteosarcoma and is a primary driver of treatment failure and poor prognosis in patients with metastatic or recurrent disease[31]. In this context, targeted silencing of PVT1 effectively removes this survival shield, lowering the apoptotic threshold and resensitizing osteosarcoma cells to doxorubicin-mediated cell death. Indeed, LNP-siPVT1 combined with doxorubicin elicits a significantly higher apoptotic rate than either monotherapy, underscoring that PVT1 inhibition represents a viable and effective strategy to overcome chemoresistance. Notably, the use of a humanized immune system model further strengthens the translational relevance of these findings, as it permits assessment of potential immune-mediated effects in a context more closely resembling human physiology.

The current study has several limitations. First, while we have demonstrated antitumor efficacy in a humanized immune system mouse model, this model relies on PBMC reconstitution and primarily reflects adaptive immune components. It does not fully recapitulate the complexity of the tumor immune microenvironment, including tumor-associated macrophages, myeloid-derived suppressor cells, and the intricate cytokine networks that characterize human osteosarcoma. Second, although we have demonstrated synergistic effects with doxorubicin and sorafenib in vitro, the in vivo combination studies were limited to doxorubicin alone. Third, our study did not systematically investigate the impact of PVT1 silencing on the circPVT1 isoform, which shares overlapping sequences and may also contribute to oncogenic functions.

In conclusion, we found that PVT1 is aberrantly overexpressed in osteosarcoma and serves as both a prognostic biomarker and a promising therapeutic target. LNP-siPVT1 exhibits favorable physicochemical properties and pH-responsive release behavior, enabling efficient and specific silencing of PVT1 in osteosarcoma cells. LNP-siPVT1 significantly suppresses cell proliferation and promotes apoptosis in vitro, and synergistically enhances the anti-tumor efficacy of doxorubicin and sorafenib. In a humanized immune system mouse xenograft model, combination treatment with LNP-siPVT1 and doxorubicin achieves superior inhibition of tumor growth without notable systemic toxicity or organ impairment. LNP-siPVT1 offers a potential combination strategy for osteosarcoma treatment.

## 5 Conclusion

Aberrant overexpression of lncRNA PVT1 is associated with malignant progression and poor prognosis in osteosarcoma, supporting its role as a valid prognostic biomarker and actionable therapeutic target. Lipid nanoparticle-encapsulated siPVT1 (LNP-siPVT1) exhibits favorable physicochemical characteristics and pH-responsive release behavior, enabling efficient and specific silencing of PVT1 in osteosarcoma cells. LNP-siPVT1 significantly suppresses cell proliferation and promotes apoptosis in vitro, and synergistically enhances the anti-tumor efficacy of doxorubicin and sorafenib. In a humanized immune system mouse xenograft model, combination treatment with LNP-siPVT1 and doxorubicin achieves superior inhibition of tumor growth without notable systemic toxicity or organ impairment. These findings demonstrate that LNP-mediated targeted silencing of oncogenic lncRNA PVT1, particularly in combination with conventional chemotherapy, represents a safe and effective strategy for osteosarcoma treatment, and provides a solid experimental basis for the clinical translation of LNP-siPVT1-based targeted therapy.

## Funding

This work was supported by grants from General Projects of Gansu Provincial Joint Scientific Research Fund (24JRRG019), Zhangye Municipal Science and Technology Plan Project (ZY2024JS29), Gansu Provincial Department of Education Industrial Support Program (2025CYZC-061), Project of the Presidents Fund Team, 10.13039/100012843Hexi University (CXTD2025019).

## CRediT authorship contribution statement

**Jiantong Wei:** Conceptualization, Data curation, Formal analysis, Investigation, Methodology, Project administration, Validation, Writing – original draft, Writing – review & editing. **Zetao Qian:** Conceptualization, Data curation, Formal analysis, Investigation, Project administration, Validation, Writing – original draft, Writing – review & editing. **Qingqing Qin:** Investigation, Validation. **Hao Chen:** Investigation, Validation. **Wenqiang Liang:** Investigation, Validation. **Ting Wang:** Investigation, Validation. **Shengwan Wang:** Investigation, Validation. **Wenji Wang:** Investigation, Validation. **Yongping Wang:** Conceptualization, Investigation, Supervision, Visualization, Writing – original draft, Writing – review & editing. **Yaohua Wang:** Conceptualization, Funding acquisition, Investigation, Supervision, Visualization, Writing – review & editing.

## Declaration of competing interests

The authors declare that they have no known competing financial interests or personal relationships that could have appeared to influence the work reported in this paper.

## Data Availability

Data will be made available on request.
